# Retinal Disease and Metabolism

**DOI:** 10.3390/life12020183

**Published:** 2022-01-27

**Authors:** Zhongjie Fu, Ayumi Usui-Ouchi, William Allen, Yohei Tomita

**Affiliations:** 1 Department of Ophthalmology, Boston Children’s Hospital, Harvard Medical School, Boston, MA 02115, USA; zhongjie.fu@childrens.harvard.edu (Z.F.); William.Allen@childrens.harvard.edu (W.A.); 2 Department of Ophthalmology, Juntendo University Urayasu Hospital, Chiba 279-0021, Japan; ausui@juntendo.ac.jp

Retinal diseases, such as diabetic retinopathy (DR), age-related macular degeneration (AMD), and retinopathy of prematurity (ROP), are some of the leading causes of blindness all over the world. Several studies have recently described metabolic changes that occur during the progression of retinal diseases. In this Collection of *Life*, we present a collection of articles dedicated to various aspects of clinic and experimental research in retinal disorders. Specifically, we highlight investigations of metabolic contributions to retinal disease development and prevention, evaluations of current drug intervention and diagnosis, and the identification of new biomarkers.

## 1. Metabolism-Related Risk Factors and Regulators

The retina is highly energetically demanding, with a very high density of mitochondria in the body [1]. However, our current knowledge of retinal metabolism is still limited. Aerobic glycolysis is essential to maintain photoreceptor health, and disturbance in glucose uptake causes retinal degeneration [2,3]. Photoreceptors also utilize lipids as energy sources, and a shortage of lipid supply leads to photoreceptor dysfunction and subretinal neovascularization [4,5]. Hyperglycemia and dyslipidemia are associated with the development and progression of retinal disorders across all age groups. Emerging evidence has shown that disturbed metabolism and unbalanced retinal redox contribute to retinal disease pathogenesis.

With the advancement of current omics technologies, our understanding of metabolic alterations in retinal disorders has increased. Tomita et al. summarized the current experimental and clinical investigations of metabolic alterations in ROP, a common complication in premature infants and the leading cause of vision loss in children [6]. Lipidomics, proteomics, and metabolomics have suggested that dysfunction in the lipid and amino acid metabolism contributes to ROP [7,8,9]. Low essential lipids such as docosahexaenoic acid (DHA) and arachidonic acid (AA), low postnatal sphingosine-a-phosphate (S1P), high blood malonylcarnitine (C3DC), as well as elevated plasma amino acids such as glycine and proline correlate with ROP [6]. Accompanying metabolic changes, decreased antioxidants, and increased oxidative stress predict severe ROP. Further validation has been partially conducted using two ROP mouse models: oxygen-induced retinopathy, which mimics late-stage proliferative ROP, and hyperglycemia-associated retinopathy, which mimics early ROP [10,11]. Nutritional supplementation, hormonal modulation, and other interventions to restore retinal metabolic and redox balance are very appealing options for the prevention and treatment of ROP. Tsang et al. reviewed and discussed the beneficial effects of some nutraceuticals on ROP [12]. These nutraceuticals, including oils such as DHA, flavonoids such as green tea and Bilberries, and many others, may exert anti-angiogenic, anti-oxidative, anti-inflammatory, and anti-apoptotic effects on ROP prevention and treatment. However, further mechanistic investigations are needed to better elucidate the action of these nutraceuticals in preventing retinal neovascularization.

Oxygen-related factors have been considered key for ROP pathogenesis. The supplementation of oxygen is crucial to prevent mortality in extremely premature infants. However, hyperoxia can suppress physiological retinal vessel growth, setting the stage for late vision-threatening proliferative ROP [13]. Therefore, promoting retinal vessel growth at the early stage may prevent severe ROP. Singh et al. found that hyperoxia downregulated Myc, a critical cell cycle regulator, and upregulated P53 proteins, thereby increasing p21 protein levels in retinal endothelial cells in vitro [14]. P21, in turn, decreased pRb levels and caused cell cycle arrest in the G1 phase. Further validation in animal models of ROP would be interesting to examine if the modulation of Myc could prevent hyperoxia-induced retinal vessel loss in vivo.

In addition to ROP, the contribution of dyslipidemia to AMD has also been well documented. Hu et al. summarized and discussed our current knowledge of apolipoprotein E (ApoE) in AMD progression [15]. ApoE functions as a plasma lipid transport protein and is involved in lipid clearance. APOE is produced and secreted locally from RPE and Müller glia and is a major contributing factor in drusen formation in AMD pathogenesis [16]. *APOE2* is associated with increased risk for AMD, while *APOE4* seems to correlate with decreased risk for AMD. APOE isoforms have shown differential behavior in their interaction with retinal microglia. This review also discusses the interaction between APOE and the complement system, amyloid-beta.

Moreover, Riddell et al. also found that there was a metabolic shift in myopia (short-sightedness) and hyperopia (long-sightedness) induced chicks at an early stage, accompanied by decreased retinal neuronal responses [17]. In the chick myopia model, increased expression of genes involved in oxidative phosphorylation and decreased expression of genes involved in the signal transduction pathways were identified with microarray analysis. Meanwhile, increased expression of genes involved in sphingolipid metabolism and decreased expression of genes involved in branch-chain amino acid degradation were found in the chick hyperopia model. Further exploration with advanced omics technologies would provide more insight into the mechanistic changes at the early hours of myopia and hyperopia induction.

Targeting unbalanced retinal redox and oxidative stress could be a therapeutic approach to treat retinal metabolic disorders. Pietras-Baczewska et al. investigated whether there was an altered status of antioxidant in the vitreous of patients with rhegmatogenous retinal detachment (RRD) with or without proliferative vitreoretinopathy (PVR), in comparison to patients with macular hole (MH) or epiretinal membrane (ERM, controls) [18]. The results showed no significant differences in total antioxidant status (TAS) and antioxidant enzymes, including superoxide dismutase and glutathione reductase. The duration of the disease influenced TAS in the vitreous of RRD with PVR. However, as the authors pointed out, a limitation of the study was that the control group patients might also experience an altered pattern of antioxidant status, as MH or ERM are retinal degenerative diseases with a long duration.

Peroxisome proliferator-activated receptors (PPARs) are strong metabolic modulators. Lee et al. summarized the therapeutic potential of PPARα in various CNS diseases such as Alzheimer’s disorder and neuropsychiatric disorders [19]. PPARα activation also modulates neuroinflammation, a significant contributor to amyotrophic lateral sclerosis and multiple sclerosis. Experimental studies have also demonstrated positive effects of PPARα activation by fenofibrate on ischemic stroke. In addition to the brain, fenofibrate is beneficial in preventing the progression of diabetic retinopathy, a common eye complication in diabetic patients. More recently, pemafibrate, which has higher potency and selectivity for PPARα than fenofibrate and exerts fewer side effects on kidney injuries, has been designed [20,21,22]. With the knowledge gained from PPARα therapeutics in CNS disorders, we can better understand the protective roles of PPARα in retinal disorders. In addition to PPARs, the liver-derived endocrine insulin-like growth factor (IGF)-1 is also a significant regulator of retinal metabolism and angiogenesis. IGF-1 stimulation of VEGFA secretion in human RPE cells has been reported [23]. Puddu et al. found that caveolin-1 was a mediator of IGF-1-induced VEGFA secretion in ARPE-19 cells [24]. Immortalized human microvascular endothelial cells (HMEC-1) under the treatment of conditional media from ARPE19 with caveolin-1 knockdown showed reduced endothelial cell migration rate, further elucidating the mechanisms behind IGF-1 regulation of VEGFA.

## 2. Evaluation for Retinal Disease Treatment

VEGFA induction is a critical contributing factor to pathological retinal angiogenesis [25]. Anti-VEGF therapy is one of the major treatments for diabetic macular edema (DME), AMD, retinal vein occlusion (RVO), and ROP [26,27]. Usui-Ouchi et al. retrospectively analyzed prognostic factors affecting a short-term response to anti-VEGF therapy in DME [28]. They presented higher glycosylated hemoglobin levels, a larger foveal avascular zone (FAZ), and a higher number of microaneurysms in the pericapillary network (PCN), which are poor anatomical prognostic factors in anti-VEGF therapy. Their data also revealed a significant correlation between the number of microaneurysms (MAs) in the PCN and FAZ size. Cystoid macular edema (CME) was the most common type of DME in poor responders, and CME retinas had a higher number of MAs in the PCN and a larger FAZ size. In addition, Tanwani et al. reported that treatment outcome based upon best-corrected visual acuity (BVCA) and central macular thickness (CMT) was the same despite different numbers of injections and intervals of injections to DME patients [29]. They summarized that despite significant differences in the number of anti-VEGF injections administered and the overall length of treatment of DME patients, all retina specialists had similar outcomes concerning the changes in BCVA and CMT. Although these data came from one practice site and a small number of patients, the study suggests that treatment protocol could be refined to reduce the number of injections in DME. On the other hand, Wicinski et al. evaluated possible modifications in blood coagulation parameters and pro-inflammatory cytokines in AMD patients treated with intravitreal injections of aflibercept (IVA), one of the anti-VEGF drugs [30]. Their results suggested that the repeated administration of IVA may influence the common blood coagulation pathway such as thrombin time (TT). They also showed that the serum concentration of IL-18, a pro-inflammatory cytokine, was significantly increased during the initial loading phase of IVA. Thus, although anti-VEGF agents are a widespread treatment worldwide, there is still room for debate regarding prognostic factors, injection method, frequency, and side effects.

Anti-VEGF therapy has also been widely used for ME in branch retinal vein occlusion (BRVO) [31]. Sakanishi et al. investigated the relationship between treatment outcomes of IVA for ME due to BRVO and subfoveal choroidal thickness (SCT) [32]. There was no significant difference in central foveal thickness improvement, visual acuity, and the number of IVA injections between the SCT non-thickened and SCT thickened groups. ME recurrence was higher in the SCT thickened group significantly. They concluded that although SCT before treatment did not affect the efficacy of IVA, the risk of ME recurrence was lower in cases in which SCT became thin during treatment. Thus, the SCT change could be a therapeutic indicator of IVA for BRVO. Although the choroidal thickness varies markedly among individuals daily and decreases with age, further large-scale studies of choroidal thickness, including the examination time, could resolve this problem.

In addition to anti-VEGF therapy, laser treatment is another option for diseases such as DME. One of the possible therapeutic mechanisms of laser treatment to the retina is the activation of metabolic states of RPE cells and subsequent functional improvement [33]. However, there is no effective way to evaluate the impacts of laser therapy on the metabolism and function of the irradiated retina and its surroundings. Sonntag et al. presented an exciting report demonstrating changes in a fluorescent lifetime (FLT) after retinal laser treatment in vivo using fluorescence lifetime imaging ophthalmoscopy (FLIO) [33]. After identifying a region around the laser spot where the FLT was transiently shorter than surrounding areas, the authors speculated that it may indicate metabolic changes in the surrounding cells responding to laser invasion. Thus, they summarized that FLIO may be a useful tool for the evaluation of the metabolic and structural response of the retina to laser treatments.

Exploring and developing a generalized approach for rare retinal disorders resulting from various genetic causes is therapeutically convenient. Retinitis pigmentosa (RP) is a hereditary disease that causes progressive photoreceptor cell loss [34]. There have been very limited clinically available drugs for RP treatment up to this point. Matsuo et al. demonstrated in vitro and in vivo evidence of the neuroprotective effect of NK-5962, which is a crucial component of photoelectric retinal prosthesis (OUReP) to prevent retinal neuronal apoptosis [34]. Intravitreal injection of NK-5962 in the eyes of RCS rats reduces the number of apoptotic cells in the photoreceptor layer. The authors suggested NK-5962 as a candidate treatment for delaying the deterioration of retinal dystrophy such as RP.

## 3. Diagnosis and Biomarkers of Retinal Disease

Early detection of retinal diseases is essential both for preventing disease progression and developing potential therapeutic targets at an early stage. The ultra-widefield fundus imaging systems, optical coherence tomography (OCT), and OCT angiography (OCTA) are now essential for diagnosing various retinal diseases [35]. Wysocka-Mincewics et al. described the prevalence of pediatric diabetic retinopathy and stressed the importance of screening in patients with type 1 diabetes [36]. In this review, the authors discussed the usefulness of the findings obtained by OCT and OCTA, including foveal thickness, foveal avascular zone (FAZ), and vascular density for the early detection of retinopathy. In addition, continuous supplementation of insulin using an insulin pump is recommended for the treatment of pediatric patients because it has dramatically reduced the incidence of early symptoms of DR in the pediatric population. Although they mentioned limitations of OCTA, such as confinement of imaging to only the central retina, not its periphery, and time requirements for good fixation, which could be problematic for young children; improvements to the device could reasonably resolve these problems. Ozawa et al. newly invented the eyelid clamper, which is able to keep an eye open without the need for topical anesthesia [37]. They quantified pixels of the imaged retina and found the system could capture fundus images of sufficient size as in the case of conventional tape fixation. These devices could aid fundus imaging in children.

The fundus photos from the health examination program are important sources for investigating the prevalence of retinal diseases and the association between retinal status and systemic condition [38]. Shimizu et al. investigated the prevalence of ERM using fundus photographs from 5042 eyes of 2552 subjects in Japan’s health examination program database [39]. ERM was detected in 275 eyes (5.5%) from 217 subjects (8.5%). By univariate analyses, ERM was significantly more common in the eyes with higher Scheie’s H grade, S grade, and glaucoma. Moreover, ERM was significantly common in subjects with more frequent histories of hypertension and hyperlipidemia, older age, shorter body height, higher systolic blood pressure, more frequent use of medication for hyperlipidemia and hypertension, and thicker intimal medial thickness. By multivariate analyses, older age was the only significant factor of ERM prevalence. The major limitation of this study was a lack of data regarding myopia and cataract surgery history, which were reported to be risk factors of ERM. In addition, ERM was diagnosed only with fundus photos without OCT.

Concentric RP is a rare and atypical RP with retinal degeneration limited in the peripheral region [40]. Nakahara et al. identified 15 patients from 14 families with concentric RP out of 673 with RP and presented their clinical characteristics and genetic analysis [41]. Compared to typical RP, patients with concentric RP had better visual acuity and preserved ellipsoid zones. Two cases had myotonic dystrophy-associated retinopathy in the cohort, an important differential diagnosis of concentric RP. Genetic testing was performed on nine patients, and mutations in the EYS gene were found in one patient, and mutations in the RP9 gene were found in another patient. As many patients are assumed to be undetected or left undiagnosed due to mild symptoms, further studies are needed to determine the difference and prevalence of concentric RP from typical RP. Matsuo et al. reported cases of bilateral swelling of the optic disc in three consecutive patients with cryopyrin-associated periodic syndrome or Blau syndrome, rare autoinflammatory diseases [42]. They described bilateral swelling of the optic disc as a plausible common ocular feature in autoinflammatory diseases if it occurred as a complication in addition to conjunctivitis, anterior uveitis, and posterior uveitis.

In addition to evaluating disease characteristics using retinal images, the quantification of various proteins or molecules in the serum, aqueous humor, and vitreous is helpful to predict the severity of various retinal or vascular diseases and understand the disease pathogenesis [7]. Low et al. demonstrated that high levels of decorin, a small-leucine-rich proteoglycan, increase with the severity of DR [43]. They also showed that non-responders to DR treatment present high aqueous humor decorin concentrations, and decorin concentrations were positively correlated with visual acuity in DR patients. They concluded that decorin levels in the aqueous humor were found to be elevated in DR subjects, possibly due to a compensatory response to changes in retinal microvasculature during hyperglycemia. Increasing the sample size may help further classify DR groups. Takayanagi et al. examined the association between serum oxidative stress markers in eyes and retinal vessel diameters with primary open-angle glaucoma to investigate the interaction between the pathogenesis of glaucoma and vessel narrowing [44]. In the POAG group, the central retinal artery equivalent (CRAE), central retinal vein equivalent (CRVE), and serum biological antioxidant potential (BAP) were lower compared to controls, and the BAP showed a significant correlation both with CRAE and systolic blood pressure. These data suggested a clinical impact of systemic antioxidant treatment for glaucoma patients.

In summary, this research topic includes original research articles, brief reports, and reviews regarding novel therapeutic approaches, evaluations of drug interventions, and mechanistic investigations of neurovascular retinopathies. These articles also elucidate the crucial contribution of retinal metabolic alterations to disease pathogenesis. We believe that this collection will be interesting to a broad audience of scientists in retinal research.

## Data Availability

Not applicable.
